# The Use of Platelet-Rich Plasma in the Treatment of Diabetic Foot Ulcers: A Scoping Review

**DOI:** 10.7759/cureus.43452

**Published:** 2023-08-14

**Authors:** Viktor Kunder, Kiran C Sharma, Zehra Rizvi, Ricardo Soubelet, Monika Ducharme

**Affiliations:** 1 Osteopathic Medicine, Nova Southeastern University Dr. Kiran C. Patel College of Osteopathic Medicine, Fort Lauderdale, USA; 2 Oral and Maxillofacial Surgery, Palm Beach Gardens Medical Center, Palm Beach Gardens, USA

**Keywords:** diabetic ulcers, non-healing ulcers, wound care, diabetic foot ulcers, prp, platelet rich plasma, platelet-rich plasma

## Abstract

Platelet-rich plasma (PRP) has been recognized as a method of treatment in medicine since the 1980s. It primarily functions by releasing cytokines and growth factors that promote wound healing; these growth-promoting factors released by PRP enact new processes such as angiogenesis, collagen deposition, and tissue formation that can change wound-healing outcomes. Many studies recognize that PRP aids in chronic wound healing, which is advantageous for patients who suffer from chronic diabetic foot ulcers (DFUs). This scoping review aims to examine the literature to identify the efficacy of PRP use in the healing of DFUs. The objective of this study is to explore whether PRP has a beneficial effect on healing completeness and the rate of healing on DFUs. Following PRISMA (Preferred Reporting Items for Systematic Reviews and Meta-Analyses) guidelines, we searched randomized-controlled trials involving PRP use in diabetic patients with foot ulcers using PubMed, Medline, CINAHL Complete, and Cochrane Database of Systematic Reviews. We restricted the search to articles published during 2005-2022, full texts in the English language, articles involving patients aged 19 years or older, articles that used PRP specifically on DFUs, articles that included a control group, and articles with human subjects. The initial search yielded 119 articles after removing duplicates. The final analysis for relevance yielded eight articles. In seven of the eight studies, the PRP group showed significant results, with either faster healing, more complete healing, or a larger percentage of healed participants. In the one study that did not give conclusive evidence of accelerated healing with PRP, PRP was used as an adjunct to fat grafting and only used once. Application styles of PRP for treatment were shown to influence the level of healing in patients, with injected PRP appearing to achieve the best results compared to topical PRP application. However, this was not conclusive due to the involvement of several other variables. Two studies additionally found PRP to be useful in healing refractory DFUs, and one study found that PRP use in patients with additional comorbidities was still more effective in healing DFUs than standard wound control. This study used scoping review methodology with randomized-controlled trials to examine the literature regarding PRP use in the healing of DFUs. The evidence suggests that PRP is a useful tool in reducing healing times and improving rates of complete wound healing in DFUs. There is room for further research in the application styles of PRP before conclusive statements can be made on the efficacy of injected versus topical PRP healing, based on the findings in this study. The results of this review provide a baseline for further research on PRP use in patients with diabetes and can be used by physicians and public health experts to guide future treatment options for DFUs.

## Introduction and background

Platelet-rich plasma (PRP) has been recognized as a method of treatment in medicine since the 1980s and has since had widespread use in many surgical fields [1]. However, despite its decades of presence in medicine, it is still not widely utilized in wound treatment and does not have a standard formula for preparation. The standard procedure to prepare homologous PRP involves drawing the patient's blood, centrifuging the blood to get multiple layers, and extracting the buffy coat that contains PRP. Many clinics use different methods of preparing PRP that range from centrifugation to varying methods of administration onto the wound site, such as topical versus injected, yet, the essential components are the same. As the name suggests, PRP is a concentration of platelets [1]. It primarily functions by releasing cytokines and growth factors (platelet-derived growth factor [PDGF], transforming growth factor-beta [TGF-B], vascular endothelial growth factor [VEGF], fibroblast growth factor [FGF], epidermal growth factor [EGF]) that stimulate cellular processes involved in tissue repair, angiogenesis, and collagen synthesis. Many studies recognize its use in chronic wound healing, such as diabetic foot ulcers (DFUs) [1-3]. 

Many patients with diabetes are predisposed to lower-extremity wounds, such as foot ulcers due to peripheral neuropathy, reduced blood circulation, and impaired immunologic response [3]. Often, refractory to standard wound care such as dressing changes and blood sugar control, these patients’ foot ulcers do not heal quickly, and they are left dealing with chronic wounds [2]. These foot ulcers are a serious complication of diabetes and can lead to worse outcomes such as amputations and increased mortality [3]. Many treatment options are available for DFUs, such as debridement, skin grafts, and revascularization [3]. PRP has been considered a promising treatment for these patients due to its release of growth-promoting factors that encourage angiogenesis, collagen deposition, and tissue formation to positively impact wound-healing outcomes. 

Poor wound healing is a known complication of uncontrolled diabetes, and these patients' predisposition for chronic foot ulcers makes investigations of chronic wound-healing treatments extremely important [2]. There are many goals of chronic wound healing, including achieving wound closure, complete healing, and rate of healing as quickly as possible [2]. This scoping review aims to examine the existing literature to identify the efficacy of PRP use in healing foot ulcers in diabetic patients.

## Review

Materials and methods

Using PRISMA (Preferred Reporting Items for Systematic Reviews and Meta-Analyses) guidelines, a comprehensive electronic search was performed to identify randomized controlled trials (RCTs) that discuss the link between the use of PRP on a non-healing DFU compared to a control group. This scoping review utilized primary studies found via a search on PubMed, Medline, CINAHL Complete, and Cochrane Database of Systematic Reviews. Studies published from 2005 to 2022 were included based on the inclusion and exclusion criteria described below. Three reviewers performed the selection process independently and blindly based on the inclusion and exclusion criteria.

Search Strategy

A computer-assisted literature search of the databases as mentioned earlier was performed to identify studies that fall under the inclusion criteria as follows: (1) articles published between 2005 and 2021, (2) full texts in the English language, (3) articles involving patients aged 19 years or older, (4) articles that used PRP on specifically DFUs, (5) articles that included a control group, and (6) articles using human subjects. Articles that did not contain a control group, treated wounds not specified on the foot, or not in diabetic populations were excluded. The search was conducted in December 2022 and yielded 119 results.

Identification of Studies

We used the following text words and search phrases in our search: ((Platelet-rich plasma) OR (Platelet rich plasma) OR (PRP) OR (PPP)) AND ((diabetic foot ulcer) OR (DFU) OR (diabetic foot ulcers) OR (DFUs)). This search was performed using the modifiers of articles published between 2005 and 2021, full texts in the English language, patients aged 19 years or older, and human subjects. 

Data Extraction

After screening and applying the inclusion criteria to the studies obtained from the databases, all researchers organized relevant information on a data log that included the author and year, sample size, treatment method, and results. The outcomes were documented on a Google Docs spreadsheet. With the information organized, a thorough discussion of each article was conducted to determine whether it was within the inclusion criteria. Disagreements were resolved through discussion.

**Figure 1:**
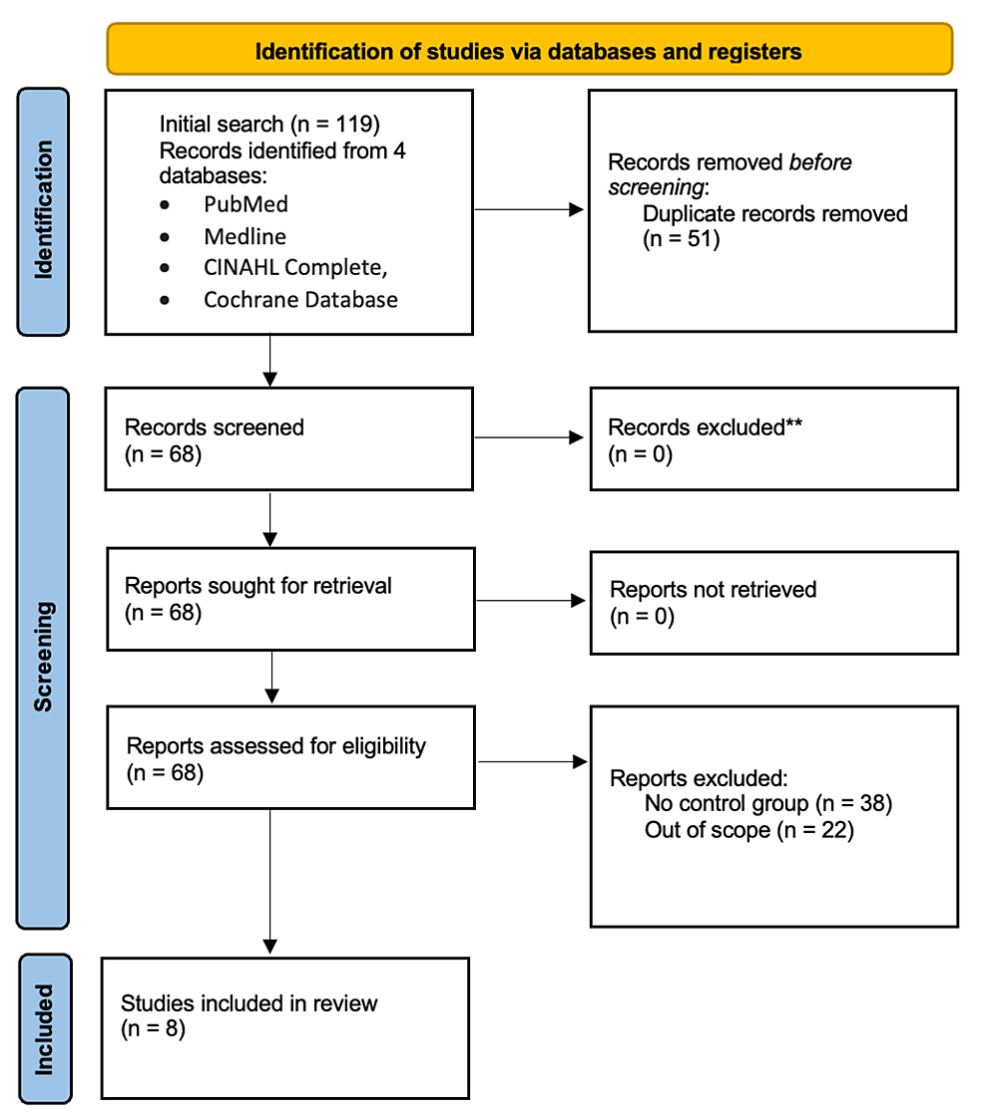
PRISMA flow diagram depicting the study selection process PRISMA: Preferred Reporting Items for Systematic Reviews and Meta-Analyses.

The initial search elicited 119 articles based on the outlined search criteria. After removing 51 duplicates, 68 articles underwent a quality assessment. Of these, eight articles qualified for this study. These articles involved RCTs that used PRP to treat non-healing DFUs (Figure 1).

Results

A total of eight studies were identified using the research selection pathway described in Figure 1. Table 1 reports the characteristics of the studies included in this scoping review, which included studies that utilized RCTs.

**Table 1:** The summary of findings of the selected studies PRP = platelet-rich plasma; PPP = platelet-poor plasma; Grade 1 healing = 100%; Grade 2 healing = 80-99%.

Study	Number of Participants	Application Type	Volume Used	Frequency	Key Findings
Malekpour et al., 2021 [3]	PRP = 47, Control = 43	Topical gel	20 mL	Once every 3-4 days for 3 weeks	Mean duration of healing: 55.6 days (PRP) and 78.6 days (control) - Smoking, gender, age, and hypertension did not affect the efficacy of PRP.
Driver et al., 2006 [4]	PRP = 19, Control = 21	Topical gel	20 mL	Every 3-4 days for 12 weeks	Percentage healing at the 12-week mark: 68.4% (PRP) and 42.9% (control)
Saad et al., 2011 [5]	PRP = 12, Control = 12	Topical gel	10 mL	Every 3-4 days for up to 20 weeks or until healed	Mean healing time: 11.5 weeks (PRP) and 17 weeks (PPP)
Li et al., 2015 [6]	PRP = 48, Control = 55	Topical gel	20-100 mL (based on wound size)	Every 2 weeks for 12 weeks	Grade 1 healing: 85.4% (PRP) and 67.3% (control). Grade 1 and 2 healing: 95.8% (PRP) and 70.9% (control). Average time to complete healing: 36 days (PRP) and 48 days (control).
Ahmed et al., 2017 [7]	PRP = 28, Control = 28	Topical gel and injection	20 mL	Every 3-4 days for 12 weeks	Complete healing: 86% (PRP) versus 68% (control). Complete wound healing achieved after 2 weeks: 29% (PRP) versus 7% (control).
Singh et al., 2018 [8]	PRP = 29, Control = 26	Injection	20 mL	Once a week until the ulcer heals	Complete healing occurred in all patients in the study group. Mean time to complete healing: 36.7 ± 3 days (PRP) compared with 60.6 ± 3.7 (control).
Elsaid et al., 2020 [9]	PRP = 12, Control = 12	Topical gel	20 mL	Once every 3-4 days for 20 weeks or until healed	25% (PRP) healed completely versus 0% (control). The remaining 75% in the PRP group showed significantly reduced ulcer dimensions compared to the control group. 41.6% of patients on PRP showed a response to treatment versus 8.3% in the control group.
Smith et al., 2020 [10]	PRP = 6, Control = 6	Unspecified	52 mL	Once on day 0	33% of patients healed from fat graft with PRP (at days 73 and 80) versus 33% with fat grafting alone (at days 78 and 80).

All studies in this review recognized PRP as useful as a wound-healing treatment for DFUs when used more than once, and multiple studies identified PRP as more efficacious in chronic wound healing than standard treatment options. In addition, most studies showed a significant difference in the mean healing times using PRP versus the control group. Of these studies, 12 weeks was adequate time for most ulcers to heal [3-9].

Several additional variables were involved in many of these studies, which provided additional data about the usage of PRP in DFUs.

PRP Application Type

Five studies used only topical PRP gel, one used both topical PRP gel and injections, one used only PRP injections, and one used PRP injections along with fat grafting. The injected PRP study showed wound healing approximately 39% more quickly than the control group [5]. Multiple studies using topical PRP application only saw wound-healing times that were approximately 25% faster [3,4,8]. All but one study showed that regardless of application type, PRP use still significantly improved healing time and overall wound outcome [3-9]. When PRP was applied only once as an adjunct treatment with a fat graft, it showed no additional benefit [10].

Frequency and Duration of Treatment

Frequency of treatment varied from only one application, to application once a week, once every two weeks, and twice a week. The studies that applied PRP twice a week showed more benefit than those that applied only PRP once or every other week [3-5,7,9]. Duration of treatment included four timeframes: only once, three weeks, 12 weeks, and 20 weeks. Applying PRP only once showed no difference from the control group [10]. There was not enough evidence to determine if applying PRP for a longer duration from the three-week study differed from the 12- or 20-week studies.

Comorbidities

All studies addressed diabetic patients. One study also considered smoking, blood pressure, and age as factors and found PRP to still increase healing outcomes compared to control groups even in groups with these additional factors [3].

Discussion

This scoping review aimed to identify the efficacy of PRP in the healing process, percentage, and rate of DFUs compared to standard treatment. Following a thorough review and analysis of the eight included RCTs, our review suggests that PRP did aid in the healing process for diabetic patients with foot ulcers; however, several factors determined the degree and rate of healing.

First, in all cases except one [10] where PRP use was pitted against a control group, the PRP group showed either faster healing [3,5,6,8], more complete healing [7,9], or a larger percentage of healed participants [4,6,7]. There were no situations in the included studies where the control group had a higher healing rate or decreased wound size compared to a group with isolated PRP-only use. Only one study [10] did not show conclusive evidence that PRP caused accelerated healing in DFUs, and this study did not have an isolated PRP variable group.

Application styles of PRP for treatment were shown to have an effect on the level of healing in patients. In one study [8] that injected PRP into the study group, all patients achieved complete healing, and healing time was approximately half of that of the control group. Most studies [3-6,9] applied PRP as a topical gel, and there was variety in these results as well. This difference in results could be attributed to having different control groups between studies, as well as the difference in the frequency of treatment. None of the topical PRP application groups achieved a similar healing time difference between their control and study groups as the injected PRP group did.

Another study [3] found that even with additional risk factors such as a history of smoking or hypertension, age, and gender, PRP use was still more effective in healing DFUs than the standard control groups in all cases.

Limitations of included studies

Although this review used the inclusion and exclusion criteria to use only quality RCTs, there were limitations within these studies. One of these limitations was that the control and standard treatment differed by hospital and region. Thus, it is difficult to compare the results of the studies with complete equivalence in instances where the control groups were fat grafting or ointment rather than standard wound care. Our comprehensive search yielded eight articles that fit our inclusion criteria so we could not standardize the control group. Additionally, the frequency and duration of treatment for the studies varied. It was difficult to accurately compare results between studies that only had one application of PRP and studies that had multiple applications per week. Additionally, some studies had a much longer timeline than others, allowing for longer data collection. Some studies continued the study until the ulcer was healed, which allowed further data to be collected on complete healing times between treatment modalities. There was very limited evidence of bias in these studies.

Limitations of the review process

Articles before 2005 were excluded, which removed earlier studies relevant to this topic. Also, applying strict inclusion and exclusion criteria may have excluded many relevant articles. For example, only RCT study types were included, removing several relevant articles. Additionally, all articles that we reviewed were in the English language and focused specifically on ulcers located on the feet in diabetics. Studies that used PRP to treat ulcers in any other region, even in diabetics, were excluded. 

Implications for research and practice

The findings of this review show that PRP is highly useful in treating DFUs, which is impactful for health providers in their treatment plans for these patients. Public health measures can be used to educate patients on this treatment option, as well as to make providers aware of the efficacy of this option. However, several studies mentioned room for further research in order to make conclusive statements about the use of PRP. This study analyzed only eight articles that met our inclusion criteria. Further, peer-reviewed research on this topic would be helpful in increasing the power of the results. For example, there is no published literature about the ideal timeline for using PRP as an adjunctive therapy. This field needs to be explored further with more RCTs to establish a better trend for the ideal timeline for treatment, as well as solidify a proper control. 

Unfortunately, many patients present with several health issues at once or an extensive past medical history. Further research can also introduce additional variables such as comorbidities to assess if PRP will have a similar effect on those patients as in patients with isolated DFUs. Additionally, research on treatment frequency and minimum timeline would supplement the current research concluding the efficacy of PRP treatment.

## Conclusions

This study used scoping review methodology with RCTs to examine the literature regarding PRP use in the healing of DFUs. The evidence from our review suggests that PRP is a useful tool in reducing healing times and improving rates of complete wound healing in DFUs. This healing effect was also shown to be seen in patients with the comorbidities of hypertension, smoking, and older age. There is room for further research in the application styles of PRP before conclusive statements can be made on the efficacy of injected versus topical PRP healing based on the findings in this study. This review was limited by the relatively small amount of published RCTs on this topic. The results of this review provide a baseline for further research on PRP use in diabetic patients and can be used by physicians and public health experts to guide future treatment options for DFUs.
